# A Ledger of Me: Personalizing Healthcare Using Blockchain Technology

**DOI:** 10.3389/fmed.2019.00171

**Published:** 2019-07-24

**Authors:** Gary Leeming, James Cunningham, John Ainsworth

**Affiliations:** Division of Informatics, Imaging and Data Sciences, Health eResearch Centre, Manchester Academic Health Science Centre, University of Manchester, Manchester, United Kingdom

**Keywords:** blockchain, health informatics, distributed ledger technologies, personal health record (PHR), digital health

## Abstract

Personal Health Records (PHRs) have the potential to give patients fine-grained, personalized and secure access to their own medical data and to enable self-management of care. Emergent trends around the use of Blockchain, or Distributed Ledger Technology, seem to offer solutions to some of the problems faced in enabling these technologies, especially to support issues consent, data exchange, and data access. We present an analysis of existing blockchain-based health record solutions and a reference architecture for a “Ledger of Me” system that extends PHR to create a new platform combining the collection and access of medical data and digital interventions with smart contracts. Our intention is to enable patient use of the data in order to support their care and to provide a strong consent mechanisms for sharing of data between different organizations and apps. Ledger of Me is based on around the principle that this combination of event-driven smart contracts, medical record data, and patient control is important for the adoption of blockchain-based solutions for the PHR. The reference architecture we present can serve as the basis of a range of future blockchain-based medical application architectures.

## Introduction

The opportunities presented by a predominantly digitized healthcare sector have begun to drive government policy and direction in the UK and elsewhere (1–3); stimulated the development of new health apps and validation needs (4); and led to advances in consumer hardware (5). Despite this, visions of overarching transformation within the sector still struggle to gain traction (6). One of the main challenges facing digitized healthcare is that of enabling data sharing, or interoperability, between applications, data sources, and systems (7). Personal Health Record (PHR), is a health record that can be accessed, and to some extent, controlled directly by the patient to whom that record belongs (8), and requires such interoperability in order to actually deliver the more personalized care their concept promises. However, the complex governance issues involved in protecting the very personal and private data that PHRs capture mean there is a clear need for more transparency in the areas of consent, anonymisation, and data ownership (9). To achieve this healthcare systems need to balance complex system change against patient safety, evidence-based practice and validation processes. Patient expectations are changing. Citizens want a health system that adapts to their lifestyle and needs in the same way that banking and airlines have, while not always being aware of the inherent differences in how healthcare works, and the specific challenges of meeting the combined needs of the legal and ethical frameworks of both care and research (10). Sharing of data, in particular, is seen as a huge challenge (11). This is not solely because of the technical limitations, which are a part of problem, but also the need to meet complex information governance rules, organizational needs and priorities, public expectations of privacy for their health data, and even a distrust between different care boundaries (12). These issues have, previously, limited data sharing and adoption of standards, and slowed the development of solutions that can be truly disruptive to the delivery of care.

Interest is growing amongst the research community in Distributed ledger technologies (DLT) and blockchain projects as solutions to these challenges (13, 14). These terms will be used interchangeably in this paper to refer to the general class of DLT. Whilst methodologies differ, most focus on the need to manage consent and permissions for the use of data (15). These solutions are often described as personal health record systems as they are designed to put patients in charge of their own data, building on blockchain as a platform built to enforce the creation of trust and the management of identity. These self-described PHR blockchain applications are now being developed using in order to test these solutions however it is still open for debate whether these solutions really fulfill the needs of a PHR, and if blockchain provides a useful platform or not. This paper summarizes current blockchain development and reviews the specific use-case of blockchain-based PHR solutions, looking at the specific questions and shortfalls that these solutions have as healthcare applications. In response to this we present a system design and reference architecture that responds to these shortfalls and presents a solution offering real transformation in digital health.

## Background

### Blockchain

A simple definition of blockchain is “an open, distributed ledger that can record transactions between two parties efficiently and in a verifiable and permanent way” (16). Traditional currencies need trusted third parties, usually governments and banks, to guarantee transactions, underwrite funds, and verify identity to prevent fraud. This can be a challenge on the internet where identity is difficult to guarantee, and so transactions cannot always be trusted. Bitcoin was conceived as the underlying technology of a peer-to-peer electronic currency (latterly “crypto-currency”) (17), providing an elegant solution to the “Byzantine Generals Problem” (18). This is the question of how to achieve consensus amongst a group of peers where some members of the group are broadcasting (potentially deliberately) incorrect information, for example to double count payments into a bank account, or present a false identity. The Bitcoin paper applied a solution to this problem in order to produce a mechanism of exchanging electronic “coins” (i.e., fungible representations of a store of value) without the need to rely on a centralized trusted third to guarantee the integrity or underlying security of the currency.

Identity on a blockchain is established via the production and ownership of a secret cryptographic key, part of a public/private key pair. Keys are used to digitally sign transactions on a blockchain. Although the ownership of the key can be considered anonymous, the identity itself is trustworthy. Transactions are recorded in time-ordered “blocks” of information, with consensus as to the canonical ordering and content of these blocks being decided on via a “proof-of-work” mechanism where so-called “miners” compete to brute-force a solution to a computationally expensive calculation (unique to each potential block on the blockchain), with miners incentivized to do this via a reward of the underlying currency being attached to the production of a correct solution. The proof-of-work mechanism serves, in place of a trusted third party, to guarantee and secure the integrity of the system. This proof-of-work mechanism has however drawn criticism due to the immense amount of energy now consumed in providing this security (40). Additional concerns exist around the quasi-anonymous nature of bitcoin transactions and its use in the facilitation of black-market transactions.

Building on the initial work to design a crypto-currency a new platform was started, called Ethereum (19). Ethereum expands on the idea of exchange of value to include the ability to run Turing-complete computer programmes, known as “smart contracts.” This extension means that rules for payment, for different types of data storage, and new models of interaction between computer software that is not on a blockchain can be created. Ethereum attaches a cost to undertake a computation based on the complexity of the contract and the amount of data to be stored, which is known as *gas*. When a transaction is completed there is an exchange of the ethereum currency, *ether*, to the agreed *gas* value which is given to the miner as well as the cost of the transaction itself. For example, management of complex supply chains and payment can now be more automated. A visitor at a restaurant can order fish and be assured as to where it was caught, by which boat, when, and that it was stored at the correct temperature using internet-enabled thermometers (20) as the data is captured and recorded through smart contracts. A range of socially minded projects have emerged from this technology, such as new models of identity for refugees in development with the UN to help people who have lost key identity papers and qualifications, and tracking pharmaceutical manufacturing and supply to reduce the potential for fraud (21). Similarly, Hyperledger (22) a distributed ledger platform developed by IBM, released under an open source license, provides a set of tools and services that are able to meet the more complex demands of using blockchains in industry sectors other than banking. These smart contract-based services derive external information through integration with so-called “oracle” services. Oracle services are application programming interfaces (APIs) that interact with software and data that is available separate to the blockchain, introducing an often required degree of explicit external trust into these systems. For example, a service that managed payment for utilization of a cloud computing service would call for further detail from the service logs of the provider for service usage in order to calculate costs.

A degree of Bitcoin's reliability and trust stems from it being hosted openly on public networks, and the transparent history of all transactions that this brings. Ethereum is also a public network but it is possible to also establish a private solution, as can Hyperledger. This can be a benefit where data needs to remain private but organizations want the advantages of immutability and identity that blockchain provides – though justification of the use of such technology in intrinsically trusting environments can be difficult. In general blockchain brings benefit where there exists at least some degree of potential mistrust between peers on a given network. Private blockchains may also forgo “proof of work” for another, less computationally expensive, method for the creation of blocks such as “proof of authority.” In proof of authority the nodes of the distributed system vote to accept a transaction, which is viable in a closed system which would prevent any single node from controlling the whole network.

With the huge transformations that digital technology has brought over the last 30 years many viewed blockchain as the next “disruptive” technology poised to rapidly transform whole sectors of business and the economy. Despite the hype, the last decade of blockchain development has seen tremendous internal growth but it has failed to be deliver transformational impact. There is now an acceptance that blockchain is less disruptive than it is foundational. Rather than providing a mechanism for replacing existing ways of working directly, as the internet has done with banking, it provides the groundwork for future technology innovation and disruption. This has been compared to network protocol that underpins the internet (23). Originally developed in the 1970s, it was over a decade before the creation of the world-wide web, and a decade more before the emergence of internet-enabled companies such as Google.

### Blockchain-Based Healthcare Solutions

Applications using blockchain in health were identified through a search for the terms “PHR,” “Personal Health Record,” “Blockchain,” “Distributed Ledger Technology” and “DLT.” This was conducted over the academic literature and the gray literature to find solutions that are in use or development. Eleven solutions were found. Solutions that did not provide a whitepaper or details as to the features of the application were discounted, with five remaining for review.

All of the solutions are in relatively early stages of development. All also focus on the perceived key benefit of blockchain to provide an audit of access to the data and to allow the patient to manage consent. Management of consent and access to healthcare data has attracted the greatest attention as a potential target for blockchain-based applications, with an emphasis on the patient being provided with the ability to define rules for access to their health data. This need has been highlighted to both health professionals and the public in the wake of privacy concerns raised through projects such as the UK National Health Service (NHS) project Care. Data (24), which attempted to create a single, centralized repository of all patient data without clear guidance, consent or controls. Table 1 lists the solutions and key DLT features.

**Table 1:** List of reviewed blockchain projects.

**Solution**	**Public token**	**Core technology**	**Public chain**	**Link**
GuardTime	No	KSI Blockchain	No	https://guardtime.com/blog/estonian-ehealth-partners-guardtime-blockchain-based-transparency
Carechain	No	Ethereum	Yes	https://www.carechain.io/files/CareChain_The_Infrastructure_Consortium.pdf
MedRec	No	Ethereum	No	https://medrec.media.mit.edu/
Dovetail	No	Hyperledger	No	https://www.dovetaillab.com/
MedicalChain	Yes	HyperLedger and Ethereum	Yes	https://medicalchain.com/en/whitepaper/

Reviewing the documentation available for each of these solutions there are five common features that can be identified and described.

**1. Health Data Is not Stored in the Chain**

All of the solutions describe security of the data as a key benefit of using blockchain technology but none store personal health data in their implementation of blockchain. For example, MedRec “does not ‘store' the record directly; rather encodes metadata that allows records to be accessed securely by patients, unifying access to data across disparate providers” (25). By only storing metadata MedRec stores basic information such as ownership and permission rather than the patient record being requested. Storing large amounts of data on a blockchain is recognized as expensive, both in financial and computational senses, due to the need to cryptographically sign data and encrypt it, to cover the token costs of data storage on a public chain and because of the replication requirements of a distributed data store. As such the blockchains do not secure the data directly but is used as a gateway, or pointer, to external data stores, such as Guardtime's proprietary implementation, which links information on how the patient records have been used with the source (26). The European Union General Data Protection Regulations (GDPR) (27) citizens have a right to be forgotten. In health this has to be balanced against other legal needs for health data but storing data within an immutable data store, even if held privately, could be challenged. The nature of the integration to ensure that the source data is not tampered with is generally not described. In one case this is because it is considered out of scope and the responsibility of the data owner (25). A suggested solution is to record a hash of the source data that is held off-chain, which can be used to check that the data has not been modified without an update to the chain (28). Storing health data on a chain also creates other potential risks, especially on public chains. For example, MedRec note that “A key issue is that, even without the direct disclosure of a patient name, inference about who a particular patient is could be drawn from metadata of one ethereum address with multiple others” (25).

**2. Audit of the Use of Data Is a Key Feature**

As health data is not stored on the chain and a key feature of the technology is as a distributed ledger it is not surprising that the ability to create an immutable audit trail is an important benefit for all of the solutions. It is recognized that medical records have a legal basis and so blockchain solutions are offered as a key way of validating that the health record has not been altered. MedicalChain note in their whitepaper that “Medical records are to be considered not only as medical documents, but also as legal documents. To pass off a rewritten record as contemporaneous is a criminal offense and any retrospective changes have to be clearly marked, dated and signed, and the reason for such changes clearly documented. Altering existing medical records, removing records, or adding false records puts a healthcare professional at risk of medicolegal repercussions” (28). Guardtime's solution implements a solution that records every update and access to an individual's medical record, which “makes it impossible for the government or doctors or anyone to cover up any changes to healthcare records and that's really powerful” (26). Working with the company Patients Know Best, who specialize in a web-based PHR app, Dovetail Labs have developed a solution to “provide a fully audited ledger of medical data interactions” and “harness[es] certain unique qualities of distributed ledger technology to verify identity, store consent and create tamper-proof audit records of every data exchange” (29).

**3. Permissions and Consent to Access Data for Care**

Consent to access a medical record for direct care is a subject that can vary in interpretation and legal requirements. In the UK the latest Caldicott report (30) suggests that consent for direct care is not needed, but this does not address the complex legal environment around the sharing of data between legal entities, whether hospitals or GP Practices, that also need to be agreed. This is usually accomplished through data sharing agreements, but there are projects such as the Great North Care Record that suggest that consent should be the basis for all sharing of records. As the concept of care also extends to private sector providers, and companies providing health and well-being services and apps, the need for clear mechanisms becomes greater. MedRec note that “Electronic Health Records (EHRs) were never designed to manage the complexities of multi-institutional, lifetime medical records. As patients move between providers, their data becomes scattered across different organizations, losing easy access to past records. As providers—not patients—are the primary stewards of EHRs, patients face significant hurdles in viewing their reports, correcting erroneous data, and distributing the information” (32).

For a patient to understand and consent to their record being used in different circumstances, for example to seek a second opinion from an overseas doctor not bound by the same information governance rules, there must be a platform for recording and managing that consent that is also independent and agnostic to the sources of data. According to CareChain solutions other than blockchain are not viable, as they require either a platform able to communicate with every different electronic health record system in a standardized, point to point way, or for there to be a single, global solution for managing patient data, which is not likely to be adopted by all (33).

Dovetail Lab also recognize this as a key use case for their solution: “Giving patients access, visibility and control over their health data using explicit and informed consent to drive data sharing delivers the right data, when and where it is needed” (29). With strong identity management and the ability to manage permissions to access data through smart contracts, such as in Ethereum, or by the built-in permission system of Hyperledger Fabric, the patient can be put in charge of their data and, in doing so, get greater visibility, and insight into the data held about them.

**4. Permissions and Consent to Access Data for Secondary and Research Uses**

Secondary uses and research based on medical record data is critical to ensuring that healthcare is delivered safely and that new insight into changes in population health can be identified and, if needed, managed and measured through large-scale intervention. Increasingly the value in real world evidence at a patient level is also recognized, for example in the GSK clinical trial in the UK, the Salford Lung Studies (34), the connected patient medical records with a safety monitoring system and case report form for advanced analysis of potential adverse events.

While the need for consent for direct care purposes can be open for discussion that question of secondary uses is clearer. Although data can be anonymised and shared, it is increasingly recognized that the risks of re-identification are such that consent is the preferred mechanism for the use of medical records beyond direct care (35). As the potential customers for this audience include large companies, such as pharmaceuticals and AI, this is identified as a potential revenue stream for blockchain platforms by allowing patients to provide permission to use the data, possibly in exchange for some form of token, backed by transparency as to how the data are being used. These tokens can have a monetary value or be used in exchange for other healthcare services. MedRec is one of the leaders in the development of this type of solution, and currently manage their tokens by giving providers “proof of authority,” as they are already trusted holders of the data. However, “to incentivize mining from providers and academic institutions, who would in turn receive anonymised, aggregate medical data that could be used in data analysis” (25). Carechain also seek to empower patients “to offer both healthcare professionals and researchers access to their entire health history as well as to directly purchase services in a global marketplace to improve their health” (33). Similarly, MedicalChain “participants will be compensated in MedTokens. Patients will be given the ability to unlock the monetary value that their health data holds, they will be more engaged with their health conditions and the next generation of cutting-edge medicine will be empowered” (28).

**5. Enabling Telehealth**

Telehealth is growth area for healthcare which also brings increased challenges for quality of care, information security and integration of data. Linking health data with provenance is important for helping both clinicians and patients understand the quality of their data and how it can inform decisions about care. For example, there is a difference in expectations and quality in data from validated medical devices as opposed to commercial fitness trackers and unvalidated health apps (31). Blockchain platforms offer an opportunity to be able to link across different quality of sources in a way that is difficult to implement in existing EHRs. MedicalChain, DoveTail, and CareChain are explicitly designed to support integration with telehealth data, linked to example use cases around decentralized, collaborative care enabled through trustworthy use of data. CareChain provide a CareConnect app that is designed to support this new care model based on blockchain for asynchronous care delivered in patient homes (33). Dovetail are working with GPs and “patients managing their care from home using blood glucose, blood pressure, and weight monitoring smart devices” (29). This is being tested with diabetic patients in the North of England. MedicalChain seeks to “minimize the privacy/security risk of Telehealth encounters” with “methods for verifying and authenticating the identities of the patient and practitioners” (28).

### Benefits of Blockchain in Health

These five features represent the current main themes of patient data and blockchain. Placing the patient at the center of their data sounds admirable, enabling them to share their data and even monetize it to support research. However, the uptake and success of any blockchain-based solution will be determined by the value it offers to its users, whether patients or clinicians, exceeding existing tools and services. Many healthcare solutions, blockchain or otherwise, fail to ask what is the real value proposition. For example, Greenhalgh notes that patients have to consider a number of trade-offs in the adoption of new technology, including direct health benefit, cost, the amount of surveillance and medicalization, as well as the work required to use them (6). If blockchain platforms are to develop health-specific implementations they will need to address these value propositions. Given the relative immaturity of distributed ledger technology it is not surprising that initial use cases are focussed on the immediate opportunities that DLT provides, but it seems that the benefits are more likely to be realized by healthcare providers than patients: Data sharing, permissions and audit are key challenges in healthcare, that national programmes such as the Local Health and Care Record Exemplars in the NHS in England looking to resolve. These are also key features of blockchain but patient focused solutions are asking patients to become brokers for their own data, when most patients have no interest in taking on such a role when then rewards are unclear. Studies by the Wellcome Trust (35) and Connected Health Cities (36) have both shown that the majority of citizens are not interested in managing their own data, but are more concerned with whether the data is able to support them in their own care and in providing a wider social benefit. Creating an uncertain token value for the use of data also seems unlikely to encourage citizens to take control of their data. The need for consent and improved tools for audit and management of health data is important, especially with the impact of the GDPR (30).

The focus of applying blockchain technologies to the consent and audit of the use of patient data is understandable but the challenge to adoption remains. Instead we should look more widely to understand what those patient value propositions, beyond consent, might be.

### Personal Health Records

Personal Health Records (PHRs) are a recognized application for patients to be able to access their own data in the management of their own health. By focussing on these requirements instead of just the immediate capability of DLT we can understand what are the features of application that will gain wider adoption by patients.

An exact definition of what constitutes a PHR often depends on the exact context of application (37). A UK Royal College of Physicians (RCP) report offers a definition of “…a digital tool that helps people to maintain their health and manage their care. It may do this by enabling them to capture their own health and care data, to communicate with health and care services, and/or to have access to their care record” (38). Key characteristics can include a solution that focuses on a particular patient or citizen group, organization-specific portals (e.g., single GP or hospital records), the integration of data from different providers for the patient, and personal health and well-being apps. Other uses can also include services for integration with personal health devices, such as activity trackers. PHRs can also be defined by key functionality, or use cases, as defined in the protocol for the Cochrane Review “Adult patient access to electronic health records” (8):

1. Access: access to health-related data.
2. Remind: preventive health maintenance reminders (e.g., screening).
3. Request: transactional services (e.g., repeat prescription, appointment booking, referral requests).
4. Communicate: bilateral messaging service (e.g., secure messaging for non-urgent medical questions and administrative concerns).
5. Share: patient self-documentation (e.g., manage medication list, approve content of clinical notes, upload blood pressure measurements, personal diary).
6. Manage disease: individualized disease management functions (e.g., individual guidelines, generation of an individual care plan).
7. Educate: general educational health-related information.

This functionality may not be available in a single PHR system but demonstrates that the role of a PHR is expected to be more than just access to and exchange of health record data and consent preferences. It must include patient-centered services and interactions in order to make a real impact on the patient's ability to manage their own care. It should be noted that the Cochrane Review protocol makes no reference to consent or data ownership. The RCP report identifies it as an area needing further research although did note a sample opinion that patient ownership of their web-based medical record in the PHR should be the default and the “Patient should then allow access according to own wishes, by individual, professional group, institutional, or situational consents” (38).

### Ledger of Me

Developing a successful blockchain-based solution will require meeting requirements that focus on adoption through value to the patient rather than solutions that focus on what is, fundamentally, making them sales people for their own data. Focusing on the patients' needs for tools that support them in being healthy will create a meaningful reason for a patient to not only use the solution, but also recommend it to others. In this way consent and sharing of data becomes a positive side-effect of patient use and control of their data, but solutions should not focus solely on the data, but on how the data are used. For example, apps are not trustworthy. They can be hacked, bought by a different company to be used to in different ways. They may not do what they say they will do because of a bug or a change in code leading to other unintentional side effects. Recommendations and compliance with a non-digital intervention, such as a medication or care plan are difficult to measure and complex interactions between the elements of the supply chain of care can be difficult to manage. These issues are hidden, often kept private, but can affect someone's health, especially in a complex environment of managing multiple conditions. A simple example is how the implementation of QRisk algorithm in a UK GP system was demonstrated to be incorrect (39). Ensuring that patients who may have been affected are identified and informed, understanding the future impact, and which version of the implementation of an algorithm is behind a score entered into a patient record are important. As algorithms become more critical to the implementation of medicine, with black box machine learning increasingly a feature of prediction and care, the ability to review and manage the supply chain of care for an individual becomes more important.

A blockchain-based PHR needs to establish smart contracts in order to communicate with a range of oracle services to cover the additional features of a PHR, which would facilitate the creation of a true “ledger of me” that would create a more interactive tool for health management and improvement, as well as facilitate better research and be a platform for app developers to create more trustworthy, validated health apps. This would enable an ecosystem of data, algorithms and artificial intelligence, and applications through a supply chain that is more transparent to the patient and their professional care team.

An example of this for a patient with type 2 diabetes might be for a solution that linked different apps used by the patient, with their clinical record along with other services such as a blood sugar monitors, weightloss programme, and an online grocery store. The Ledger of Me blockchain would be composed of smart contracts linking these oracle services, via their APIs, to produce a complete record across the patient's life with links to help manage algorithm lifecycles through permissions to use the data for validation and future research. Building a record across the use and mix of algorithms, and other interventions, will allow for a richer description of the outcomes of different interventions in real-world settings. This requires an understanding of more than just the data that is recorded in a medical record, to create a richly-woven, immutable chain of patient activity.

Solutions such as MedRec have already described how telehealth can be linked to a blockchain record. However, in order to provide value further benefits of linking smart contracts with, for example, reminders to get blood tests and use an internet-enabled blood sugar monitor could be configured and recorded on the ledger, and then confirm that the test results have been received and are within range. The ability to map between recommendations, for the clinician and the patient, with an immutable, and provable, record of the event would be invaluable to measure both quality and efficacy of interventions. Recording this in an independent, decentralized platform reduces the opportunity for errors and fraud while enabling the patient at the center of their care, independent of an individual provider software solution. When requesting an appointment or a prescription the request and fulfillment of the request would also need to be logged, with the same benefits and ease as reminders. Read receipts for messages between the patient and their care team would be logged, making communication more transparent across the different services. The patient would have fine-grained control over use and access to their data, which would allow them to share not only their clinical record but also data collected in different apps and settings, for example their current weight via electronic scales or a food diary app. This sharing can be managed on a per app basis, similar to current services but with the additional security and immutability provided by blockchain to ensure that any collection of use of the data is because it is necessary and proportionate, validated by smart contracts, rather than wholesale collection of data to the benefit solely of the service or company.

Other oracle services could monitor the ledger and compare activity against an ideal care pathway that has been designed for the patient to help them better self-manage their disease, with appropriate input and support from both their care team and family. Prompts to access and record education material, for example dietary information, could also be helpful in ensuring that it is fit for purpose, and provided at the right time, rather than relying on internet articles. As all of this is recorded in the ledger of me it will support the patient to be more in control not only of their data but also their condition, and allow doctors and research teams greater insight into how the patient is using and interacting with a variety of services, and the impact of this on their condition. This in turn can be analyzed, in an anonymous manner, using machine learning to create new modes of guidance generation and validation by official bodies in the UK such as National Institute for Clinical Excellence (NICE) and the Medicines and Healthcare products Regulatory Agency (MHRA).

In building a platform to manage the “Ledger of Me” on the blockchain there are technical challenges. Blockchain is not a suitable technology to store large amounts of data due to the cost and speed of writing data, and the public nature of blockchain records. Pointers to data held within oracle services can be held but can also create a risk of accidentally revealing private information if, for example, a person was using pregnancy app this could become known to a prospective employer who may then choose to not offer a job. While illegal this may be difficult to prove and so any solution must be able to hold information securely, in a way that is only accessible by the patient or applications the patient has given permission to. There also need to be assurances of how oracle services store the data to ensure that information is not changed. This could be done through cryptographic hashes, for example. There is also a need to support key management, a critical part of proving identity, in way that also protects the data and prevents inappropriate access. Smart contract-based blockchain solutions, such as Ethereum, do however provide sufficient scope for implementing more complex key-management solutions.

### Reference Architecture

In this section we present a reference architecture describing the core features, entities and functionalities of a Ledger of Me system. This reference architecture describes the features such a system should or must have. Given the novelty and rapidly changing nature of blockchain technologies it is inevitable that significant changes to some of the key technologies will take place in the near future. For example, the Ethereum network is currently undergoing a potential shift from a proof-of-work consensus mechanism, to a so-called proof-of-stake mechanism (whereby nodes offer a potentially irretrievable stake of currency in exchange for guaranteeing arriving at a consensus view of transactions with other members of the network). As such we present a reference architecture with a view to abstracting core features of a necessary design from a particular implementation. Further, we would envisage that a ledger-of-me system would be open in the sense that multiple, distinctly implemented systems, conforming to this general architecture could exist side-by-side, potentially necessitated by differing legal or regulatory environments, or simply as a byproduct of market competition.

This openness of using the ledger-of-me platform to link different systems would also support use to support Health Information Exchange and related components such as a master patient index or a record locator service. This would allow for a more complex history and linked identity for a patient to be managed in a way that cannot be tampered with, and so be a trusted mechanism for multi-organizational record management.

The Ledger of Me system is blockchain based. Information about the core models of the system are stored on chain. This ensures two things: an immutable history of actions that has taken place is recorded and a hashing mechanism, used for verifying the presence or absence of actions. The core functionality of the Ledger of Me architecture is based around the principle of Apps interacting with Data belonging to a Patient. The Patient can *grant* and *revoke* access to Data by Apps. Hashes describing the Interaction between an App and a Patient are recorded on a Distributed Ledger. Patients can *view* and *verify* the details of the Interaction which are otherwise private.

### Models

**Figure 1:**
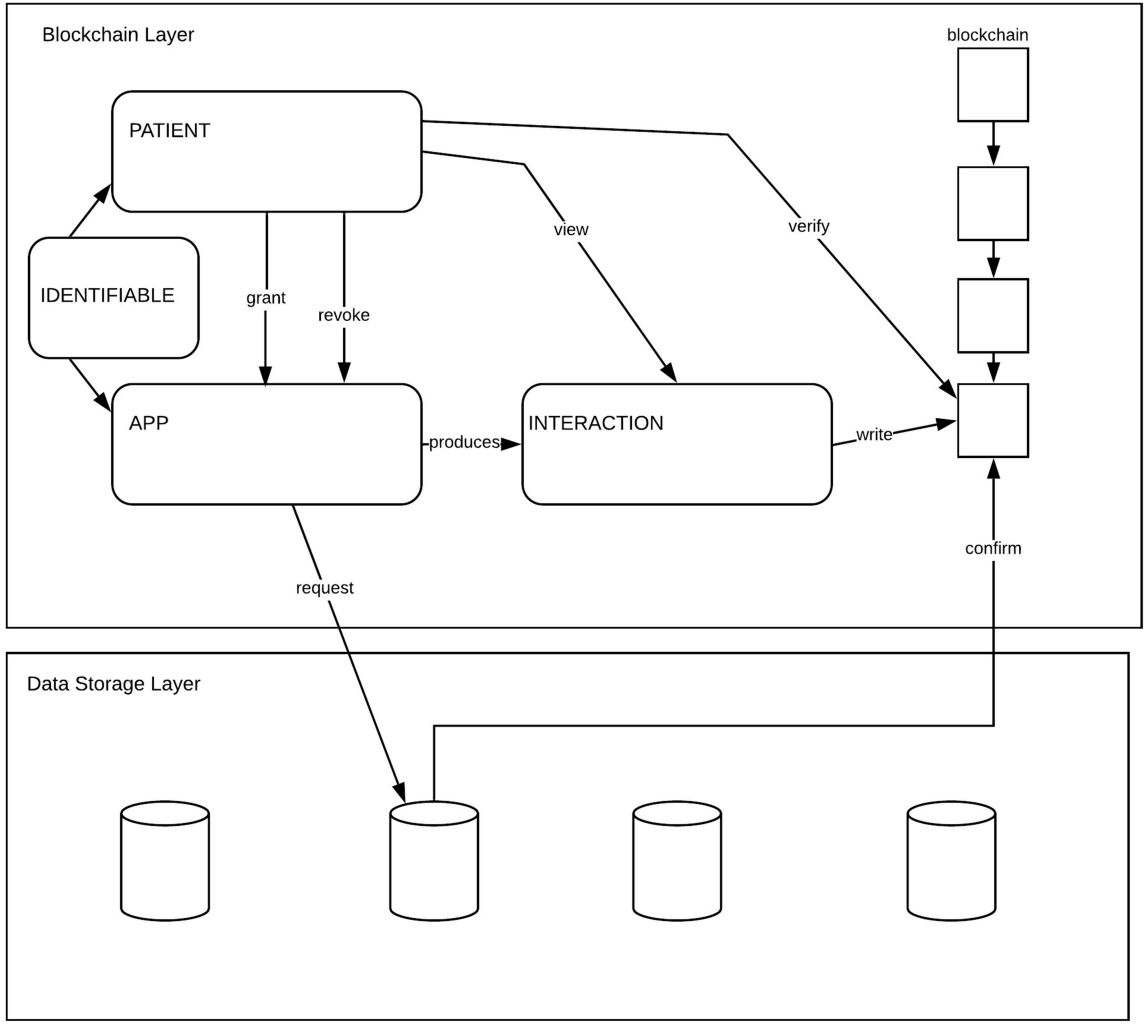
Overview of the reference architecture.

Here we outline the models that are represented in the system. These are shown diagrammatically in Figure 1 with the components of the model described below.

### Identity

An identity must contain a unique reference to a system artifact. Identities are used to distinguish between and make reference to Patients and Apps.

### Patient

A Patient is Identifiable. The Identity of a Patient relates to a user of the system. The identity of a Patient must be relatable (uniquely) to an external source of information. The identifier representing a patient must be pseudonomous in the sense that they should not be directly relatable or identifiable against externally held patient information (for example, be an NHS number) or carry personally identifiable information in the identifier itself (e.g., incorporate a person's name). A Patient carries a public key pair used to encrypt and hash data and meta-information. The private key should only be accessible by a patient and should be retrievable from a secure backup location if necessary.

### App

An App is Identifiable. The identity of Apps should be approved by a controlling third-party, with unidentified Apps being excluded from use within a Ledger of Me system.

### Interaction

An Interaction captures an action that has taken place and records the outcome of that transaction. An interaction is either between a Patient and an App or between an App and Data. It records:

- Who it is about
- Where is the interaction output stored
- What is the type of interaction
- Value of the interaction.

An interaction is stored as a cryptographic hash of the information that the interaction describes and must be recorded on the chain in an way that can only be validated a Patient key.

### Data

Data is represented in the system as pointers that reference external data sources. When information about data is recorded by an app it should be written out to a relevant external data source and a pointer to that information stored within the ledger.

### Actions

Actions are performed by the entities in the system on other entities or components of the systems. This section describes the actions that can take place.

*Grant* - A Patient may grant an App permission to read or write data. Permissions that can be granted should be granular enough that Patients can grant access to subsets of data specific to a particular application area.

*Revoke* - A Patient must be able to revoke any permission that has previously been granted. Revocation of a permission must ensure that any action by an App that was previously granted and then revoked must be denied if the action is performed at a later point in time than the revocation.

*View* - A Patient must be able to view information about Interactions that have taken place by Apps on behalf of that Patient.

*Verify* - A Patient must be able to verify that an interaction has taken place. This can be done by comparing a hash of the expected information against the hash stored within an Interaction on the chain.

*Request* - An App can request access to a data store. The request can either be to read or write data. It is the responsibility of the data store to confirm that the request is valid and that the correct information is returned to the App.

*Confirm* -A data store can confirm that a data access request is valid by searching on-chain for a relevant interaction containing a granting of permission by a Patient to an App to access that data.

### Storage and Access

Storage and access of the non-data aspects of the model are stored within a blockchain mechanism. This ensures that all records of interactions between Patients and Apps are open, auditable, immutable and transparent. Data is stored on-chain as pointers to external data sources that reside, conceptually, in the data storage layer addressing on one of the fundamental concerns of blockchain technology—the fact that data is permanently persisted when stored on chain. We make an assumption here regarding the fundamental security and integrity of off-chain data, namely that any third-party system storing such off-chain data does so in a secure and trustworthy manner.

### Blockchain Layer

The blockchain layer contains the core models of the architecture, as described above, and access to a blockchain ledger for storing the information. The ability to query the blockchain for historical transaction information regarding interactions between patients and apps must be exposed in this layer.

### Data Storage Layer

The data storage layer provides an interface for the ledger of me system, via the components of the blockchain layer, to access the data stores exposed in this layer. Data stores must expose an interface allowing them to be requested for information and for that information to be returned, securely, to the requesting application.

### Discussion: Ledger of Me as PHR

The Ledger of Me acts as a connector and record of activities between different events generated through applications which serve as different components enabling PHR functionality. Each of the key requirements for PHR (Access, Remind, Request, Communicate, Share, Manage Disease, Educate) are supported through the creation of the interaction object, linking the data, the source application, patient and clinicians through a defined data structure to be able to link the patient record within the external data store to an action. The ability to manage the creation and viewing of the transactions is provided through a Ledger of Me app that has a role in managing patient identity and cryptographic keys in a way similar to existing blockchain wallet tools. This app also allows the patient to directly interact with the data, to confirm it as valid, to provide or consent to secure, anonymous access by researchers and others, as well as manage the apps and services that have permission to interact with the ledger on behalf of the patient. In this way the patient is supported and gains value from the management of their data as a side-effect of managing their interactions with health and care providers.

The value of the Ledger of Me also extends beyond PHR to support wider healthcare improvement opportunities. The approach of managing interactions between patients and services could be applied to solving challenges with, for example, care pathway management. Creating a record of decisions made by caregivers, clinicians and the patients on a blockchain could be then composed as a smart contract-based care plan. The would allow for programmatic triggers for interventions that can be agreed and automatically activated to reduce gaps in care or missed opportunities that would be logged in an immutable record on the blockchain. In particular, the gaps and challenges or managing care across multiple organizations could be minimized, with quasi-legal agreements for services and payments, to support the link between the Ledger of Me as a personal health record and a means to action and engage with health and care providers, as well as more consumer-driven care.

## Conclusions

The review of current PHR definitions and the assessment of multiple blockchain implementations demonstrate that blockchain technology can meet the key requirements for management of consent and tracking the use of private health data. However, as the digital health domain extends to include more detailed monitoring across our lifetimes, with genetic medicine an increasingly important source of data about us, and the linkage across a wide range of health and well-being applications, such as FitBit, there is an explosive growth in the richness of data about us that will need to be managed in a way that is frictionless to the citizen. AI and algorithms are going to become an increasingly important component of healthcare. The richness of data capture will extend beyond just health services but into well-being, into personally selected consumer applications and devices which will capture long term information about you.

The quality and interaction of different service offerings and algorithms needs to be understood as a supply chain of data and care. The implementation of smart contracts offers a mechanism that can meet the wider requirements of a complete PHR as an outline of our health, across our whole lifetime, in a way that is secure and private while providing transparency of activity and control. Managing this complex supply chain requires a ledger of me that can leverage key benefits of blockchain, such as decentralized ownership and strong identity and privacy, to enable new models for supporting patients and professionals to manage care.

Our analysis of existing applications has lead us to develop a reference architecture that can be used as an underlying core of blockchain enabled PHRs. The development of this architecture stemmed from an analysis of existing implementations and differs significantly from these in that it places an emphasis on recording only meta-information about the entities and interactions present within the system on the blockchain, contrasted with a more standard approach of attempting to host, in its entirety, the PHR on the chain. Moving forwards adoption of this approach can lead to the development of successful PHR based applications that utilize the underlying privacy features present in blockchain technology.

## Author Contributions

GL led the writing of the paper, including the core of the review of the existing blockchain solutions. JC lead on the design and writing of the technical architecture with design and input from JA and GL. All authors drafted, edited, and contributed to the design of the paper.

### Conflict of Interest Statement

The authors declare that the research was conducted in the absence of any commercial or financial relationships that could be construed as a potential conflict of interest.
